# Supplementary material for: Patients with Kawasaki Disease Have Significantly Low Aerobic Metabolism Capacity and Peak Exercise Load Capacity during Adolescence
Source: Int J Environ Res Public Health. 2020 Nov 11;17(22):8352. doi: 10.3390/ijerph17228352 (PMC7696143; doi:10.3390/ijerph17228352)
Supplement: Supplementary file 1 [file ijerph-17-08352-s001.zip › Table S1.docx]

**Table S1.** Results of CPET for participants with or without CAA

|  | KD + CAA  (n=24) | KD + no CAA  (n=26) | Control group  (n=30) | *p* |
| --- | --- | --- | --- | --- |
| FVC (L) | 3.44 ± 0.74 | 3.67 ± 0.88 | 3.34 ± 0.74 | 0.286 |
| FVC% (%) | 89.18 ± 12.89 | 92.37 ± 10.47 | 88.22 ± 11.95 | 0.402 |
| FEV1 (L) | 3.05 ± 0.63 | 3.36 ± 0.76 | 3.00 ± 0.66 | 0.120 |
| FEV1% (%) | 94.75 ± 13.53 | 100.05 ± 9.88 | 93.96 ± 14.19 | 0.171 |
| FEV1/FVC (%) | 89.08 ± 5.86 | 91.86 ± 5.02 | 89.96 ± 8.42 | 0.326 |
| VO_2_/kg at AT (ml/min/kg) | 25.45 ± 5.62 | 23.73 ± 4.93 | 26.27 ± 7.53 | 0.310 |
| AT% (%) | 57.93 ± 11.09 | 56.50 ± 11.82 | 65.36 ± 16.45 | 0.036* |
| VO_2_/kg at peak (ml/min/kg) | 34.03 ± 6.60 | 33.26 ± 6.39 | 35.20 ± 9.32 | 0.635 |
| Peak% (%) | 78.38 ± 16.53 | 79.38 ± 16.56 | 86.83 ± 17.15 | 0.127 |
| Peak% exceeded 85% (Yes/No) | 9/15 | 7/19 | 16/14 | 0.126 |
| Peak O_2_ pulse (ml/beat) | 11.35 ± 2.71 | 11.01 ± 3.06 | 11.05 ± 3.46 | 0.917 |
| O_2_ pulse% (%) | 89.38 ± 16.43 | 90.69 ± 14.83 | 92.27 ± 22.28 | 0.847 |
| RER at peak | 1.17 ± 0.07 | 1.19 ± 0.10 | 1.20 ± 0.12 | 0.327 |
| PRPP | 30874.67 ± 3208.03 | 31476.12 ± 4851.24 | 31261.03 ± 4279.49 | 0.877 |
| Values are expressed as mean ± standard deviation  One way analysis of variance was used to compare differences between the three groups. | | | | |
| CPET: Cardiopulmonary exercise test; CAA: Coronary artery aneurysm; KD: Kawasaki disease; FVC: Forced vital capacity; FVC%: Percentage of FVC compared with predicted FVC; FEV1: Forced expiratory volume in one second; FEV1%: Percentage of FEV1 compared with predicted FEV1; VO_2_: Oxygen uptake; AT: Anaerobic threshold; AT%: Percentage of VO_2_/kg at AT compared with predicted peak VO_2_/kg; Peak%: Percentage of VO_2_/kg at peak compared with predicted peak VO_2_/kg; O_2_ pulse%: Percentage of peak O_2_ pulse compared with predicted peak O_2_ pulse; RER: Respiratory exchange ratio; PRPP: Peak Rate-Pressure Product  **p* < 0.05 | | | | |
