# Supplementary material for: Patients with Kawasaki Disease Have Significantly Low Aerobic Metabolism Capacity and Peak Exercise Load Capacity during Adolescence
Source: Int J Environ Res Public Health. 2020 Nov 11;17(22):8352. doi: 10.3390/ijerph17228352 (PMC7696143; doi:10.3390/ijerph17228352)
Supplement: Supplementary file 1 [file ijerph-17-08352-s001.zip › Table S2.docx]

**Table S2.** Results of CPET for participants with or without aspirin use

|  | KD group with aspirin  (n=18) | KD group without aspirin  (n=32) | Control group  (n=30) | *p* |
| --- | --- | --- | --- | --- |
| FVC (L) | 3.52 ± 0.90 | 3.58 ± 0.78 | 3.34 ± 0.74 | 0.475 |
| FVC% (%) | 87.02 ± 8.88 | 92.99 ± 12.62 | 88.22 ± 11.95 | 0.143 |
| FEV1 (L) | 3.17 ± 0.77 | 3.23 ± 0.68 | 3.00 ± 0.66 | 0.406 |
| FEV1% (%) | 93.48 ± 10.03 | 99.78 ± 12.49 | 93.96 ± 14.19 | 0.123 |
| FEV1/FVC (%) | 90.69 ± 5.54 | 90.44 ± 5.66 | 89.96 ± 8.42 | 0.930 |
| VO_2_/kg at AT (ml/min/kg) | 24.02 ± 4.11 | 24.87 ± 5.89 | 26.27 ± 7.53 | 0.448 |
| AT% (%) | 56.74 ± 11.04 | 57.44 ± 11.74 | 65.36 ± 16.45 | 0.038* |
| VO_2_/kg at peak (ml/min/kg) | 32.49 ± 6.24 | 34.27 ± 6.55 | 35.20 ± 9.32 | 0.497 |
| Peak% (%) | 77.28 ± 18.06 | 79.81 ± 15.59 | 86.83 ± 17.15 | 0.114 |
| Peak% exceeded 85% (Yes/No) | 7/11 | 15/17 | 20/10 | 0.125 |
| Peak O_2_ pulse (ml/beat) | 10.92 ± 2.82 | 11.32 ± 2.93 | 11.05 ± 3.46 | 0.897 |
| O_2_ pulse% (%) | 85.94 ± 14.87 | 92.38 ± 15.54 | 92.27 ± 22.28 | 0.431 |
| RER at peak | 1.16 ± 0.10 | 1.19 ± 0.08 | 1.20 ± 0.12 | 0.498 |
| PRPP | 31809.78 ± 4108.25 | 30837.34 ± 4141.71 | 31261.03 ± 4279.49 | 0.732 |
| Values are expressed as mean ± standard deviation  One way analysis of variance was used to compare differences between the three groups. | | | | |
| CPET: Cardiopulmonary exercise test; KD: Kawasaki disease; FVC: Forced vital capacity; FVC%: Percentage of FVC compared with predicted FVC; FEV1: Forced expiratory volume in one second; FEV1%: Percentage of FEV1 compared with predicted FEV1; VO_2_: Oxygen uptake; AT: Anaerobic threshold; AT%: Percentage of VO_2_/kg at AT compared with predicted peak VO_2_/kg; Peak%: Percentage of VO_2_/kg at peak compared with predicted peak VO_2_/kg; O_2_ pulse%: Percentage of peak O_2_ pulse compared with predicted peak O_2_ pulse; RER: Respiratory exchange ratio; PRPP: Peak Rate-Pressure Product  **p* < 0.05 | | | | |
